# Vanillic acid and methoxyhydroquinone production from guaiacyl units and related aromatic compounds using *Aspergillus niger* cell factories

**DOI:** 10.1186/s12934-021-01643-x

**Published:** 2021-08-03

**Authors:** Ronnie J. M. Lubbers, Adiphol Dilokpimol, Paula A. Nousiainen, Răzvan C. Cioc, Jaap Visser, Pieter C. A. Bruijnincx, Ronald P. de Vries

**Affiliations:** 1 Fungal Physiology, Westerdijk Fungal Biodiversity Institute & Fungal Molecular Physiology, Utrecht University, Uppsalalaan 8, 3584CT Utrecht, The Netherlands; 2 Department of Chemistry, University of Helsinki, A. I. Virtasen Aukio 1, P.O. Box 55, 00014 Helsinki, Finland; 3 Organic Chemistry and Catalysis, Debye Institute for Nanomaterials Science, Utrecht University, Universiteitsweg 99, 3584 CG Utrecht, The Netherlands

**Keywords:** 4-Hydroxy-6-methoxy-6-oxohexa-2,4-dienoic acid, 4-Oxo-monomethyl adipate, Coniferyl alcohol, Ferulic acid, Fungal cell factory, Lignin, Vanillin, Veratic acid

## Abstract

**Background:**

The aromatic compounds vanillin and vanillic acid are important fragrances used in the food, beverage, cosmetic and pharmaceutical industries. Currently, most aromatic compounds used in products are chemically synthesized, while only a small percentage is extracted from natural sources. The metabolism of vanillin and vanillic acid has been studied for decades in microorganisms and many studies have been conducted that showed that both can be produced from ferulic acid using bacteria. In contrast, the degradation of vanillin and vanillic acid by fungi is poorly studied and no genes involved in this metabolic pathway have been identified. In this study, we aimed to clarify this metabolic pathway in *Aspergillus niger* and identify the genes involved.

**Results:**

Using whole-genome transcriptome data, four genes involved in vanillin and vanillic acid metabolism were identified. These include vanillin dehydrogenase (*vdhA*), vanillic acid hydroxylase (*vhyA*), and two genes encoding novel enzymes, which function as methoxyhydroquinone 1,2-dioxygenase (*mhdA*) and 4-oxo-monomethyl adipate esterase (*omeA*). Deletion of these genes in *A. niger* confirmed their role in aromatic metabolism and the enzymatic activities of these enzymes were verified. In addition, we demonstrated that *mhdA* and *vhyA* deletion mutants can be used as fungal cell factories for the accumulation of vanillic acid and methoxyhydroquinone from guaiacyl lignin units and related aromatic compounds.

**Conclusions:**

This study provides new insights into the fungal aromatic metabolic pathways involved in the degradation of guaiacyl units and related aromatic compounds. The identification of the involved genes unlocks new potential for engineering aromatic compound-producing fungal cell factories.

**Supplementary Information:**

The online version contains supplementary material available at 10.1186/s12934-021-01643-x.

## Introduction

Aromatic compounds, such as vanillin and vanillic acid, are important flavor and fragrance compounds, and are used in the food, beverage, cosmetic and pharmaceutical industries [1–3]. Vanillin and its derivatives, vanillic acid and methoxyhydroquinone, can also be used in the production of polymers, such as epoxy resins [4–8]. Currently, less than 1% of the produced vanillin is derived from natural sources, while the majority of vanillin is obtained through chemical synthesis, mainly from guaiacol [1, 7]. Vanillin obtained through chemical synthesis is considered “artificial” based on European regulations. Therefore, new strategies and methods are being developed to obtain “natural” vanillin through biosynthesis using microorganisms [9, 10].

The metabolism of vanillin and its derivatives by microorganisms, especially bacteria, has been well studied [11]. Vanillin is toxic in low concentrations for many microorganisms and therefore the ability to degrade vanillin is essential for microorganisms that live in natural habitats or are used in industrial processes where vanillin is present in significant amounts [12, 13]. Several vanillin metabolic pathways have been described in microorganisms [11]. In bacteria, vanillin is converted to vanillic acid and this reaction is catalyzed by vanillin dehydrogenase, followed by decarboxylation to guaiacol by vanillic acid decarboxylase [14]. In addition to oxidative routes, vanillic acid can also be demethylated to protocatechuic acid by vanillate-*o*-demethylase oxidoreductase or hydroxylated to methoxyhydroquinone by vanillate hydroxylase [15–17]. The latter appears to be uncommon for bacteria [17]. All these conversions have also been observed in filamentous fungi, but in contrast bacteria the conversion towards methoxyhydroquinone appears to be common in fungi [15].

Due to the extensive study of the vanillin metabolic pathway in bacteria, many vanillin-producing bacterial systems have been described [9, 18]. However, only one strategy producing a considerable amount of vanillin using filamentous fungi has been reported [10, 19, 20]. In this method *Aspergillus niger* is used to convert ferulic acid to vanillic acid, which is further converted by *Pycnoporus cinnabarinus* to vanillin. However, this method is not efficient since both fungi can also convert vanillic acid to methoxyhydroquinone. In order to engineer efficient fungal cell factories, the metabolic pathway genes need to be identified. Vanillate hydroxylase from the fungi *Phanerochaete chrysosporium* and *Sporotichum pulverulentum* has been characterized, but the gene encoding vanillate hydroxylase remains to be identified [21–23].

It has been shown that *A. niger* converts coniferyl alcohol to ferulic acid, which is then converted to vanillic acid and further to methoxyhydroquinone [15, 24–26]. Recently, we observed that ferulic acid was converted through the CoA-dependent β-oxidative pathway to vanillic acid [26]. Deletion of the CoA-dependent β-oxidative genes did not result in abolished growth on ferulic acid. Therefore, it is possible that another pathway is present in *A. niger* in which vanillin is an intermediate [24, 25]. In this study, we aimed to identify the genes involved in the vanillin and vanillic acid metabolic pathway of *A. niger.* Whole-genome transcriptome data of *A. niger* N402 upon transfer to coniferyl alcohol, ferulic acid, vanillic acid and veratric acid was used to identify genes involved in the vanillic acid metabolic pathway. With the obtained knowledge we created two fungal cell factories that can produce vanillic acid and methoxyhydroquinone from guaiacyl lignin units and related aromatic compounds.

## Results

### Identification of candidate genes encoding putative vanillin dehydrogenase (*vdhA*), vanillate hydroxylase (*vhyA*), methoxyhydroquinone 1,2-dioxygenase (*mhdA*) and 4-oxo-monomethyl adipate esterase (*omeA*)

To identify candidate genes involved in the metabolism of ferulic acid and vanillic acid, whole-genome transcriptome data of *A. niger* N402 grown on coniferyl alcohol, vanillic acid and veratric acid were generated. Veratric acid was selected since it was observed to be converted through the same pathway as vanillic acid [11, 27]. Whole-genome transcriptome data from *A. niger* N402 grown on ferulic acid and no carbon source was obtained from Lubbers et al. [25]. Transcriptome data from *A. niger* N402 grown in minimal media (MM) without carbon source was used as control.

**Fig. 1:**
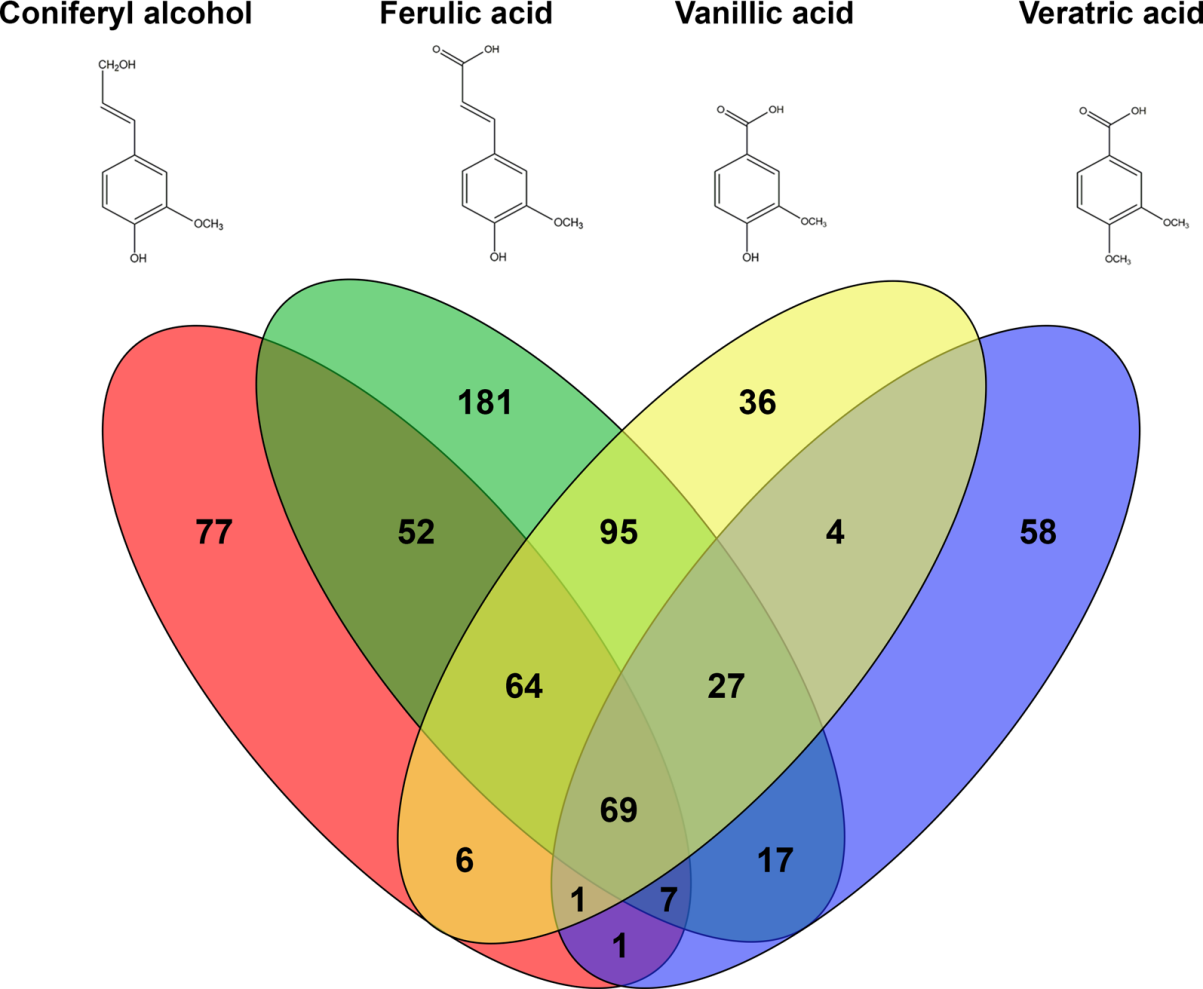
Venn diagram of the induced genes of *A. niger* N402 grown on coniferyl alcohol, ferulic acid, vanillic acid and vanillin for 2 h. Numbers represent the number of induced genes (FPKM ≥ 10, fold change (log2) ≥ 2, compared to the no carbon source control, *p*-value ≤ 0.01) of coniferyl alcohol (red), ferulic acid (green), vanillic acid (yellow) and veratric acid (blue)

**Fig. 2:**
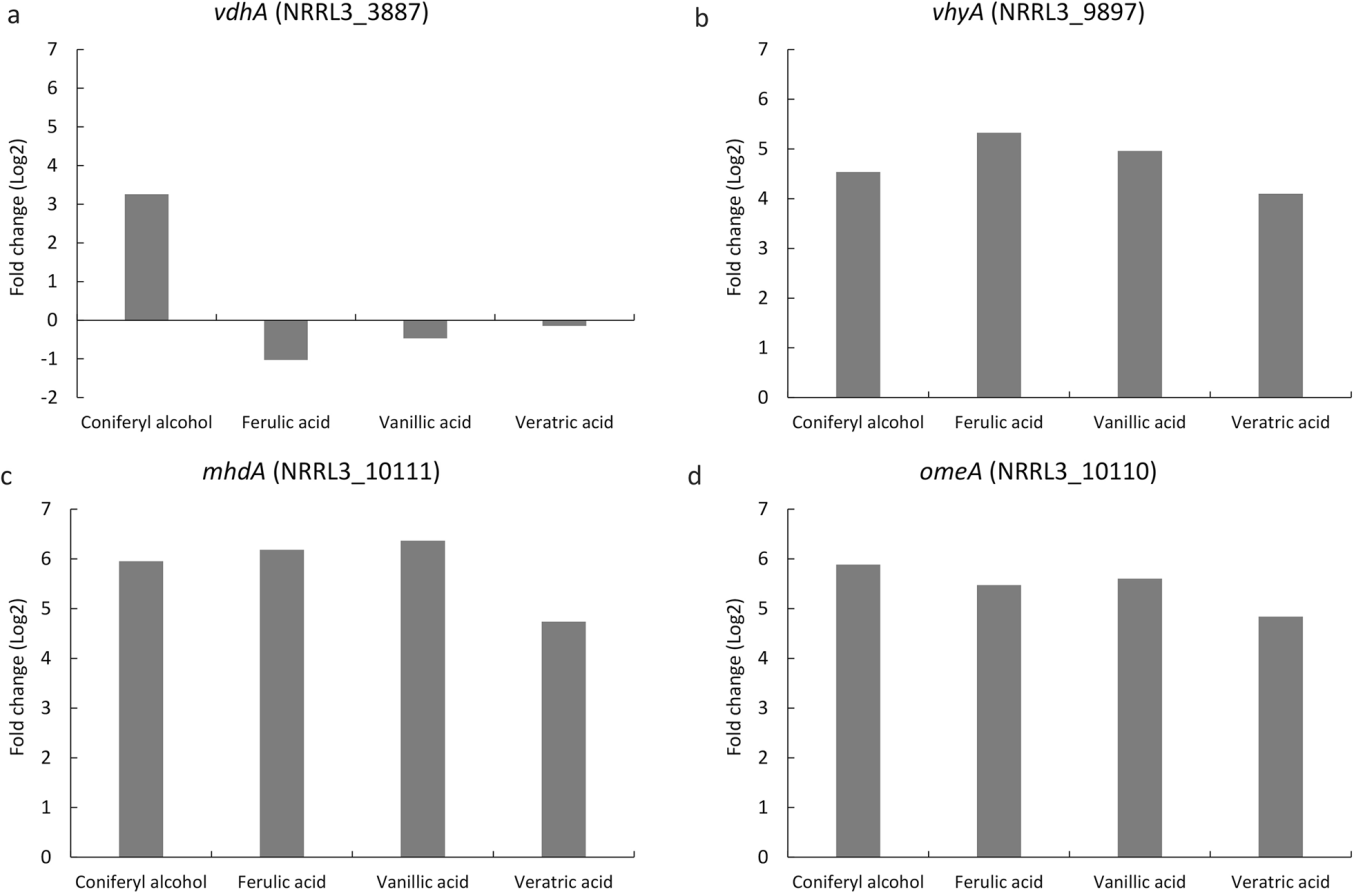
Fold change (log2) compared to a no carbon source control of the DESeq2 value of the expression of **a** the putative *vdhA* (NRRL3_3887), **b** the putative *vhyA* (NRRL3_9897), **c** the putative *mhdA* (NRRL3_10111) and **d** the putative *omeA* (NRRL3_10110) from *A. niger*. *A. niger* N402 was grown on coniferyl alcohol, ferulic acid, vanillic acid and veratric acid for 2 h

In total, 69 genes were upregulated (FPKM ≥ 10, fold change (log2) ≥ 2, *p*-value ≤ 0.01) in all conditions compared to the no carbon source control (Fig. 1), but no genes annotated as aldehyde dehydrogenases were upregulated in all four tested conditions. As no vanillin dehydrogenase has been identified in fungi, we performed a BLASTP analysis using the amino acid sequence of vanillin dehydrogenase (Vdh) from *Pseudomonas* sp. (Uniprot O05619) [28] as a query against the *A. niger* genome, resulting in 29 hits. A cut-off E-value of e ≤ − 60 was used to remove insignificant hits resulting in three genes (NRRL3_10496, NRRL3_6772 and NRRL3_3887). Only NRRL3_3887 was upregulated by coniferyl alcohol, while the other two were not upregulated in the tested conditions. Therefore, NRRL3_3887 was selected as a putative *vdhA* (Fig. 2a).

Allthough no vanillate hydroxylase-coding genes have been identified, a vanillate hydroxylase was partially characterized in *S. pulverulentum* showing that this enzyme needs FAD and NADH or NADPH as cofactor [22]. Therefore, we selected FAD-binding domain containing genes upregulated in coniferyl alcohol, ferulic acid and vanillic acid. In our dataset, only one FAD-binding domain containing gene (NRRL3_9897), annotated as a putative salicylate hydroxylase, was upregulated in all conditions and was selected as a putative *vhyA* (Fig. 2b).

Within the top 10 upregulated genes, a gene annotated as homogentisate 1,2-dioxygenase (NRRL3_10111) was upregulated under all tested conditions (Fig. 2c) and was selected as a putative *mhdA*. In addition, we observed that a putative carboxylesterase gene (NRRL3_10110) neighboring *mhdA*, was also highly upregulated under the same conditions (Fig. 2d) and was selected as a putative 4-oxo-monomethyl adipate esterase (*omeA*).

### Deletion of the putative *vdhA*, *vhyA mhdA* and *omeA* genes result in reduced growth on vanillin and its metabolites

**Fig. 3:**
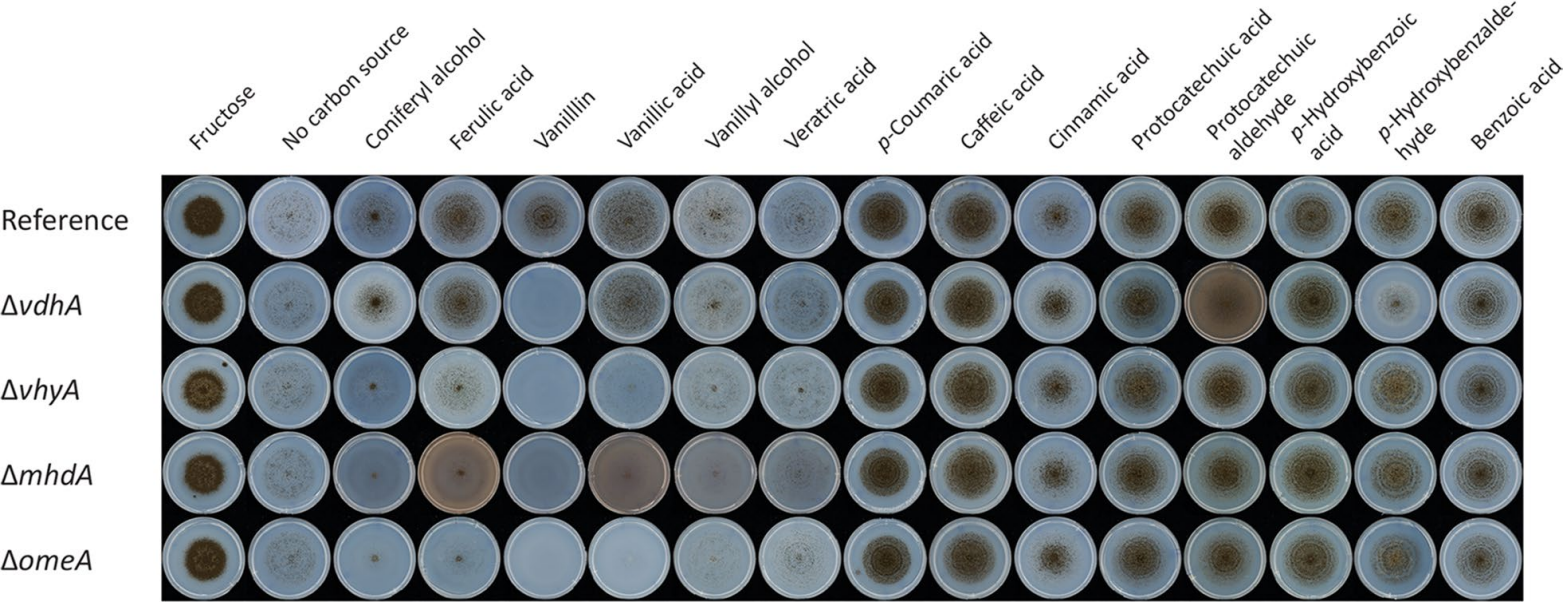
Growth profile of the *A. niger* deletion mutants Δ*vdhA*, Δ*vhyA*, Δ*mhdA* and Δ*omeA*, and the reference strain. Growth was examined on selected aromatic compounds as sole carbon source. Agar plates were incubated at 30 °C for 10 days

To verify if the candidate genes encode enzymes involved in the vanillin and vanillic acid metabolic pathway, deletion mutants of the candidate genes were made and screened for phenotypes on 14 aromatic compounds. Deletion of *vdhA* resulted in abolished growth on vanillin indicating that this gene is involved in the conversion of vanillin (Fig. 3). In addition, reduced growth on *p*-hydroxybenzaldehyde and protocatechuic aldehyde was also observed. Deletion of *vhyA, mhdA* and *omeA* resulted in reduced growth on coniferyl alcohol, ferulic acid and vanillic acid and abolished growth on vanillin, indicating that *vhyA, mhdA* and *omeA* are involved in the metabolic pathway. In addition, deletion of *mhdA* resulted in a red-brownish coloration of the medium when grown on ferulic acid and vanillic acid, which indicates that a compound, presumably methoxyhydroquinone, accumulates. After 10 days of growth, Δ*mhdA* appears to have slightly reduced growth and spore formation on vanillyl alcohol and veratric acid (Fig. 3). In addition, a light red-brownish coloration in the media was observed when grown on vanillyl alcohol indicating that it is converted towards methoxyhydroquinone. Deletion of *vhyA, mhdA* or *omeA* did not result in a phenotype on benzoic acid, caffeic acid, cinnamic acid, *p-*coumaric acid, *p-*hydroxybenzoic acid, *p-*hydroxybenzaldehyde, protocatechuic aldehyde or protocatechuic acid, indicating that these genes are not involved in the conversion of these aromatic compounds.

### Biochemical confirmation of the function of VdhA, VhyA and MhdA

**Fig. 4:**
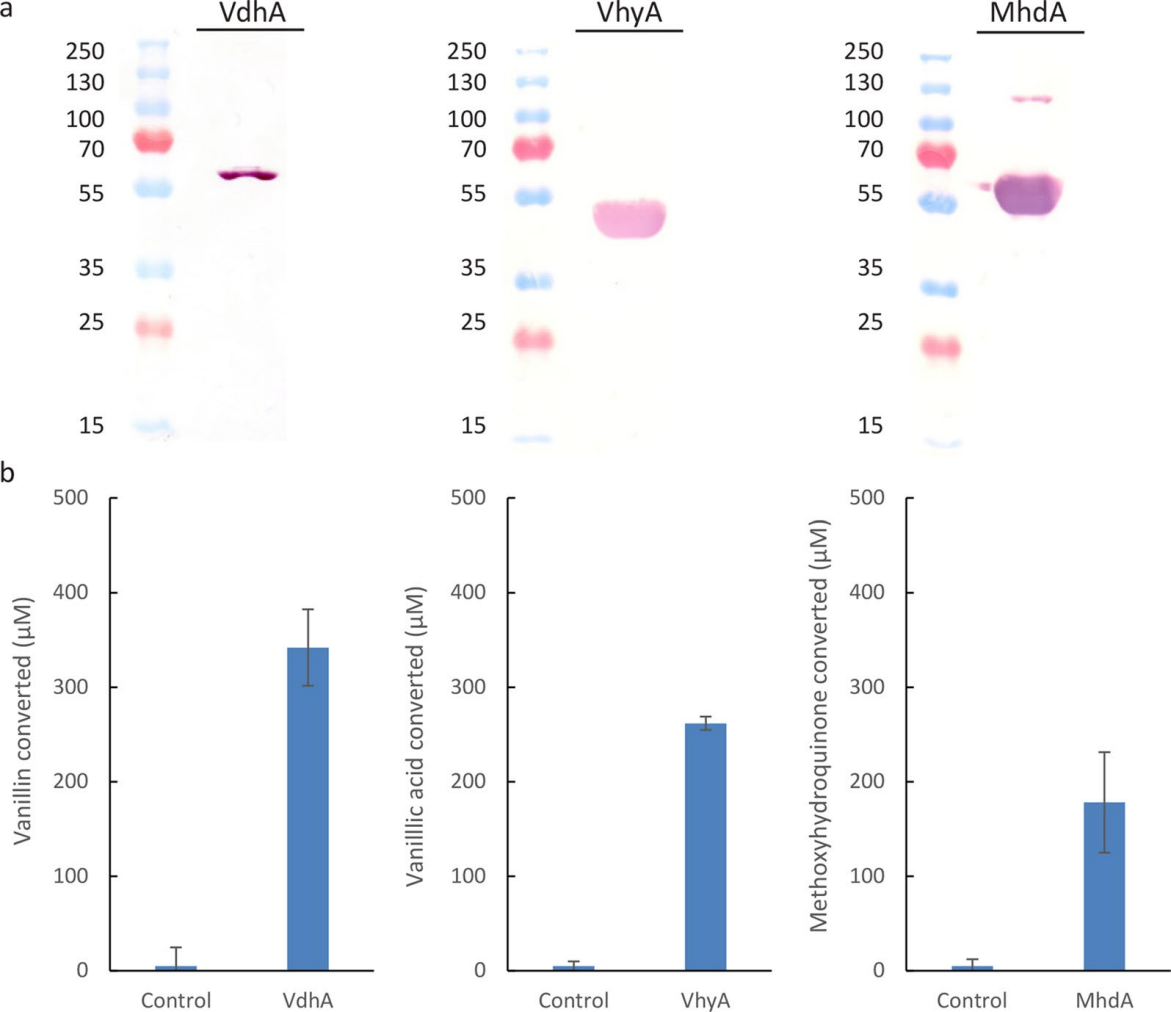
Visualization of VdhA, VhyA and MhdA by Western blot (**a**) and enzymatic activity (**b**). All enzymatic reactions were incubated for 1 h at 30 °C

To confirm the enzymatic function of VdhA, VhyA and MhdA, *E. coli* strains that produce VdhA, VhyA or MhdA were created. All three enzymes were isolated, purified, and visualized on SDS-PAGE by Coomassie brilliant blue staining (Additional file 1: Fig. S1) and Western blotting using a monoclonal antibody raised against the histidine-tag (Fig. 4a). The detected size of VhyA-His and MhdA-His corresponds with the expected masses of 48.1 and 55.5 kDa, respectively. However, the expected mass of VdhA (52.2 kDa) did not correspond with the slightly higher mass observed (Fig. 4a). Assays were performed and analyzed using HPLC, demonstrating that all enzymes had activity on their corresponding substrate (Fig. 4b).

**Fig. 5:**
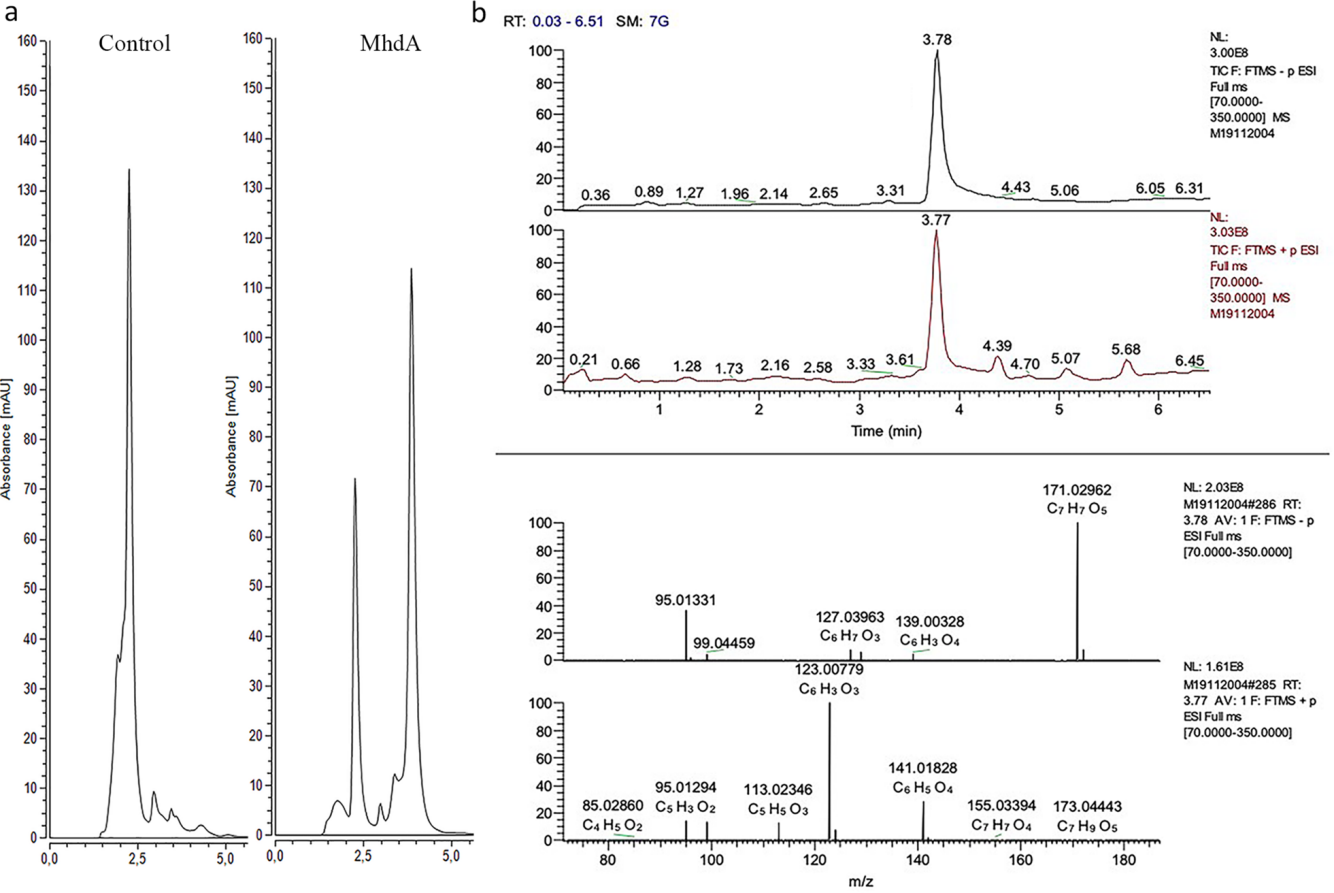
Analysis of MhdA activity by HPLC and identification of 4-hydroxy-6-methoxy-6-oxohexa-2,4-dienoic acid by Orbitrap LC–HRMS product. **a** Chromatogram of methoxyhydroquinone incubated with and without MhdA. The reaction mixture was incubated at 30 °C for 1 h. **b** Total ion chromatograms (TIC) of the ethyl acetate extracted reaction mixture of methoxyhydroquinone incubated with MhdA and the mass spectra of product 4-hydroxy-6-methoxy-6-oxohexa-2,4-dienoic acid peak at retention time 3.78 min at negative and positive ionization mode

The mixture containing MhdA showed a clear reduction in methoxyhydroquinone content and the formation of an unknown compound was detected (Fig. 5a). This compound was identified as 4-hydroxy-6-methoxy-6-oxohexa-2,4-dienoic acid using Orbitrap LC–HRMS. Identification of the detected compounds was carried out with Orbitrap Fusion high-resolution MS with both protonated and deprotonated molecules (Fig. 5b). The product peak at 3.76 min retention time was consistent with the expected product 4-hydroxy-6-methoxy-6-oxohexa-2,4-dienoic acid which showed an *m/z* [M−H]^−^ ion at 171.02962 Da (C_7_H_7_O_5_^−^, calc.171.02990 Da) and [M−H]^+^ 173.04443 Da (C_7_H_9_O_5_^+^, calc. 173.04445 Da) with below 3 ppm accuracy (1.6 and 0.1 ppm, respectively). In the positive total ion current (TIC) of the reaction mixture another unknown product peak was also detected at 2.1 min retention time that could be attributed to the addition product of imidazole (from enzyme purification) to 4-hydroxy-6-methoxy-6-oxohexa-2,4-dienoic acid with *m/z* [M−H]^+^ ion at 241.08191 Da (C_10_H_13_N_2_O_5_^+^, calc. 241.08190 Da, accuracy of 0.04 ppm), and is possibly an artifact of 4-hydroxy-6-methoxy-6-oxohexa-2,4-dienoic acid and HEPES.

### Deletion mutants accumulate vanillic acid, methoxyhydroquinone and 4-oxo-monomethyl adipate

To evaluate if the ∆*mhdA* deletion mutant can accumulate methoxyhydroquinone, an accumulation experiment in liquid media with 5 mmol/L substrate was performed. In addition, ∆*vdhA*, ∆*vhyA* and ∆*omeA* deletion mutants were also tested for the accumulation of (aromatic) compounds*.* After 24 h incubation, supernatants were collected and analyzed with HPLC (Table 1, Additional file 1: Fig. S2). The starting substrates (coniferyl alcohol, ferulic acid, vanillic acid and vanillin) were not detected in the supernatant of the reference strain indicating that these compounds were fully consumed within 24 h. Veratric acid and veratryl alcohol appeared to be slowly consumed compared to the other tested substrates.

**Table 1:** Accumulation of aromatic compounds by ∆*vdhA*, ∆*vhyA,* ∆*mhdA* and ∆*omeA* after 24 h of incubation

Starting substrate	Detected products	Reference	∆*vdhA*	∆*vhy*A	∆*mhdA*	∆*omeA*
		Conc. (mmol/L)	Conc. (mmol/L)	Conc. (mmol/L)	Conc. (mmol/L)	Conc. (mmol/L)
Coniferyl alcohol	Vanillic acid	–	–	4.78 ± 0.07	–	–
	Methoxyhydroquinone	–	–	–	3.67 ± 0.14	–
	Unknown compound^a^	–	–	–	–	Detected
Ferulic acid	Vanillic acid	–	–	4.80 ± 0.09	–	–
	Methoxyhydroquinone	–	–	–	3.93 ± 0.12	–
	Unknown compound^a^	–	–	–	–	Detected
Vanillyl alcohol	Vanillyl alcohol	1.79 ± 0.37	1.24 ± 0.25	2.13 ± 0.15	2.75 ± 0.14	0.63 ± 0.32
	Vanillin	–	0.08 ± 0.04	–	–	0.02 ± 0.04
	Vanillic acid	–	–	2.80 ± 0.06	–	–
	Methoxyhydroquinone	–	–	–	3.13 ± 0.13	–
	Unknown compound^a^	–	–	–	–	Detected
Vanillin	Vanillin	–	4.02 ± 0.13	2.98 ± 0.75	0.29 ± 0.37	0.02 ± 0.04
	Vanillyl alcohol	0.23 ± 0.05	0.64 ± 0.17	0.48 ± 0.09	–	0.49 ± 0.26
	Vanillic acid	–	–	1.58 ± 0.71	0.07 ± 0.00	–
	Methoxyhydroquinone	–	–	–	3.39 ± 1.28	–
	Unknown compound^a^	–	–	–	–	Detected
Vanillic acid	Vanillic acid	–	–	4.82 ± 0.19	0.80 ± 0.89	–
	Methoxyhydroquinone	–	–	–	4.04 ± 0.41	–
	Unknown compound^a^	–	–	–	–	Detected
Veratryl alcohol	Veratryl alcohol	3.83 ± 0.34	3.73 ± 0.38	4.04 ± 0.18	3.76 ± 0.44	3.61 ± 0.67
	Veratric acid	0.09 ± 0.02	0.65 ± 0.18	–	0.16 ± 0.07	0.08 ± 0.03
	Vanillic acid	–	–	1.11 ± 0.05	–	–
	Methoxyhydroquinone	–	–	–	2.11 ± 0.32	–
	Unknown compound^a^	–	–	–	–	Detected
Veratric aldehyde	Veratric aldehyde	0.62 ± 0.02	2.00 ± 0.19	0.76 ± 0.00	0.75 ± 0.05	0.70 ± 0.05
	Veratric acid	0.17 ± 0.13	0.40 ± 0.04	–	0.39 ± 0.13	0.18 ± 0.10
	Vanillic acid	–	–	3.48 ± 0.04	–	–
	Methoxyhydroquinone	–	–	–	3.27 ± 0.30	
	Unknown compound^a^	–	–	–	–	Detected
Veratric acid	Veratric acid	3.95 ± 1.06	4.27 ± 1.28	3.87 ± 1.14	1.51 ± 0.71	2.89 ± 1.24
	Vanillic acid	–	–	2.39 ± 0.95	–	–
	Methoxyhydroquinone	–	–	–	2.43 ± 0.54	–
	Unknown compound^a^	–	–	–	–	Detected

The starting substrate concentration was 5 mmol/L. Mean values and standard deviations were calculated from three biological replicates

^a^Unknown compound with a retention time of 4.39

Deletion of *vdhA* did not result in the accumulation of vanillin when grown on coniferyl alcohol or ferulic acid, which indicates these compounds are not converted to vanillin. Compared to the reference, vanillin consumption by ∆*vdhA* was clearly reduced (Table 1). However, a small amount of vanillyl alcohol was detected, which indicates that vanillin can also be converted to vanillyl alcohol. Trace amounts of vanillin were detected when ∆*vdhA* was grown on vanillyl alcohol, indicating that vanillyl alcohol can be reduced to vanillin.

Deletion of *vhyA* resulted in the accumulation of vanillic acid when grown on coniferyl alcohol, ferulic acid, vanillin, vanillyl alcohol, veratryl alcohol, veratric aldehyde and veratric acid, whereas vanillic acid itself was not consumed. Similar results were observed for ∆*mdhA*, which resulted in the accumulation of methoxyhydroquinone on these compounds and vanillic acid (Table 1, Additional file 1: Fig. S2, Additional file 2: Table S1). This indicates that the tested compounds are converted to vanillic acid and then further to methoxyhydroquinone.

Deletion of *omeA* resulted in the accumulation of an unknown product when grown on the tested compounds (Table 1). To investigate the accumulated product of ∆*omeA*, the unknown compound was extracted with ethyl acetate from the supernatant of ∆*omeA* grown on vanillic acid. The ^1^H-NMR of the accumulated compound corresponded well with 4-oxo-monomethyl adipate (6-methoxy-4,6-dioxohexanoic acid) [29].

### Phylogenetic study of VdhA and MhdA

To evaluate the presence of VdhA and MhdA in other fungi, a phylogenetic analysis was performed for each enzyme using selected ascomycete and basidiomycete genomes. VhyA was not analyzed since it was already included in the phylogenetic tree made for protocatechuic acid hydroxylase (PhyA, NRRL3_4659) [30].

Many aldehyde dehydrogenases were obtained as BLAST hits, but only a few clustered with *A. niger* VdhA (Additional file 1: Fig. S3). Homologs were observed in the genomes of *Aspergillus japonicus*, *Aspergillus nidulans*, *Aspergillus oryzae*, *Aspergillus fumigatus, Phaeomoniella chlamydospora* and *Talaromyces stipitatus*. Most fungi contained two or three genes annotated as homogentisate 1,2-dioxygenases (Additional file 1: Fig. S4). Some exceptions were found in *A. nidulans* and *Penicillium subrubescens*, which both contained four homologs, whereas *Trichoderma reesei* contained only one homolog. MhdA homologs were found in several Eurotiomycetes (*A. japonicus*, *A. nidulans*, *A. oryzae*, *A. fumigatus*, *P. subrubescens*, *P. chrysogenum* and *T. stipitatus*)*,* the Sordariomycete *Fusarium graminearum*, and the Dothideomycete *Cochliobolus lunatus*. All these fungi had a homolog clustering with the homogentisate 1,2-dioxygenases (HmgA) of *A. nidulans*. In addition, *Magnaporthe oryzae, Myceliophthora thermophila*, *Neurospora crassa*, *Podospora anserina*, *T. reesei* and several other fungi had a HmgA homolog. *Mucor circinelloides, Rhizopus delemar* and *Ustilago maydis* HmgA homologs clustered with the homogentisate 1,2-dioxygenase from *Arabidopsis thaliana, Homo sapiens* and *Pseudomonas putida*.

## Discussion

In this study, we identified four genes involved in the degradation of guaiacyl units of lignin (G-units), such as ferulic acid, vanillin and vanillic acid. In addition, we observed the conversion of methoxyhydroquinone to 4-hydroxy-6-methoxy-6-oxohexa-2,4-dienoic acid, which was not described before. Next to that, we demonstrated that the deletion mutants of this study can be used as cell factories for the production of vanillic acid and methoxyhydroquinone.

Vanillate hydroxylase activity has been observed in many ascomycetes and basidiomycetes and is suggested to play a major role in the degradation of the lignin G-units [31–35]. The identification of *vhyA* is an important finding which can unlock new strategies to engineer efficient fungal cell factories. For example, vanillin can be produced from ferulic acid by fungi using a two-step bioconversion process. In this process, ferulic acid or ferulic acid derived from sugar beet pulp and rice bran oil, was converted to vanillic acid by *A. niger* and was further reduced to vanillin by *P. cinnabarinus* [10, 19, 20, 36]. However, a majority of the vanillic acid was lost in this process, because it is converted to methoxyhydroquinone by both fungi. Blocking the hydroxylation of vanillic acid to methoxyhydroquinone by deleting *vhyA* in both species could improve the vanillin yield in this process. It has been shown that VhyA is closely related to PhyA [30] which is involved in hydroxylation of protocatechuic acid to hydroxyquinol. In addition, PhyA is also involved in the degradation of tannic acid and gallic acid [37]. However, it was suggested that gallic acid is converted to 2-carboxy-*cis*,*cis*-muconate by PhyA, which does not correspond with the function observed for these enzymes [38]. Therefore, it is more likely that PhyA converts gallic acid to 1,2,3,5-tetrahydroxybenzene. Despite this, it is possible that VhyA is also involved in the degradation of other aromatic compounds that were not tested in our growth assay.

Deletion of *vdhA* abolishes growth on vanillin and results in reduced growth on *p-*hydroxybenzaldehyde and protocatechuic aldehyde, indicating that *vdhA* is also involved in the benzoic acid metabolic pathway [30, 39]. It has been shown that bacterial vanillin dehydrogenases were also able to convert *p-*hydroxybenzaldehyde and protocatechuic aldehyde [40–42]. Deletion of *vdhA* did not result in reduced growth on coniferyl alcohol or ferulic acid, which indicates that these aromatic compounds are not converted to vanillin, while deletion of *vhyA* results in reduced growth on ferulic acid and coniferyl alcohol. This shows that both ferulic acid and coniferyl alcohol are converted towards vanillic acid, as previously observed [24–26], and revealed that vanillin is not an intermediate of the ferulic acid metabolic pathway. Deletion of the β-oxidative pathway genes did not result in abolished growth on ferulic acid [26]. A possible explanation is that alternative CoA-dependent β-oxidative genes are present in *A. niger*. In addition, the transcriptome data showed that *vdhA* was not induced by ferulic acid, but by coniferyl alcohol. It is possible that VdhA plays a role in the conversion of coniferyl aldehyde to ferulic acid. However, deletion of *vdhA* did not result in reduced growth on coniferyl alcohol, suggesting that other enzymes are also involved in this conversion.

Deletion of *mhdA* results in reduced growth on coniferyl alcohol, ferulic acid, vanillin and vanillic acid. In addition, deletion of *mhdA* resulted in accumulation of methoxyhydroquinone when grown on coniferyl alcohol, ferulic acid, vanillyl alcohol, vanillin, vanillic acid, veratryl alcohol, veratric aldehyde and veratric acid. Methoxyhydroquinone can be used as a building block to create epoxy resins or the thermoplastic poly(arylene ether sulfone) [5, 7, 43]. The enzyme assay with MhdA verified that the ring of methoxyhydroquinone is cleaved to 4-hydroxy-6-methoxy-6-oxohexa-2,4-dienoic acid, a reaction that has not been described before. At this moment, there are no descriptions of 4-hydroxy-6-methoxy-6-oxohexa-2,4-dienoic acid or its tautomer in literature and therefore the applications of this compounds are unexplored.

**Fig. 6:**
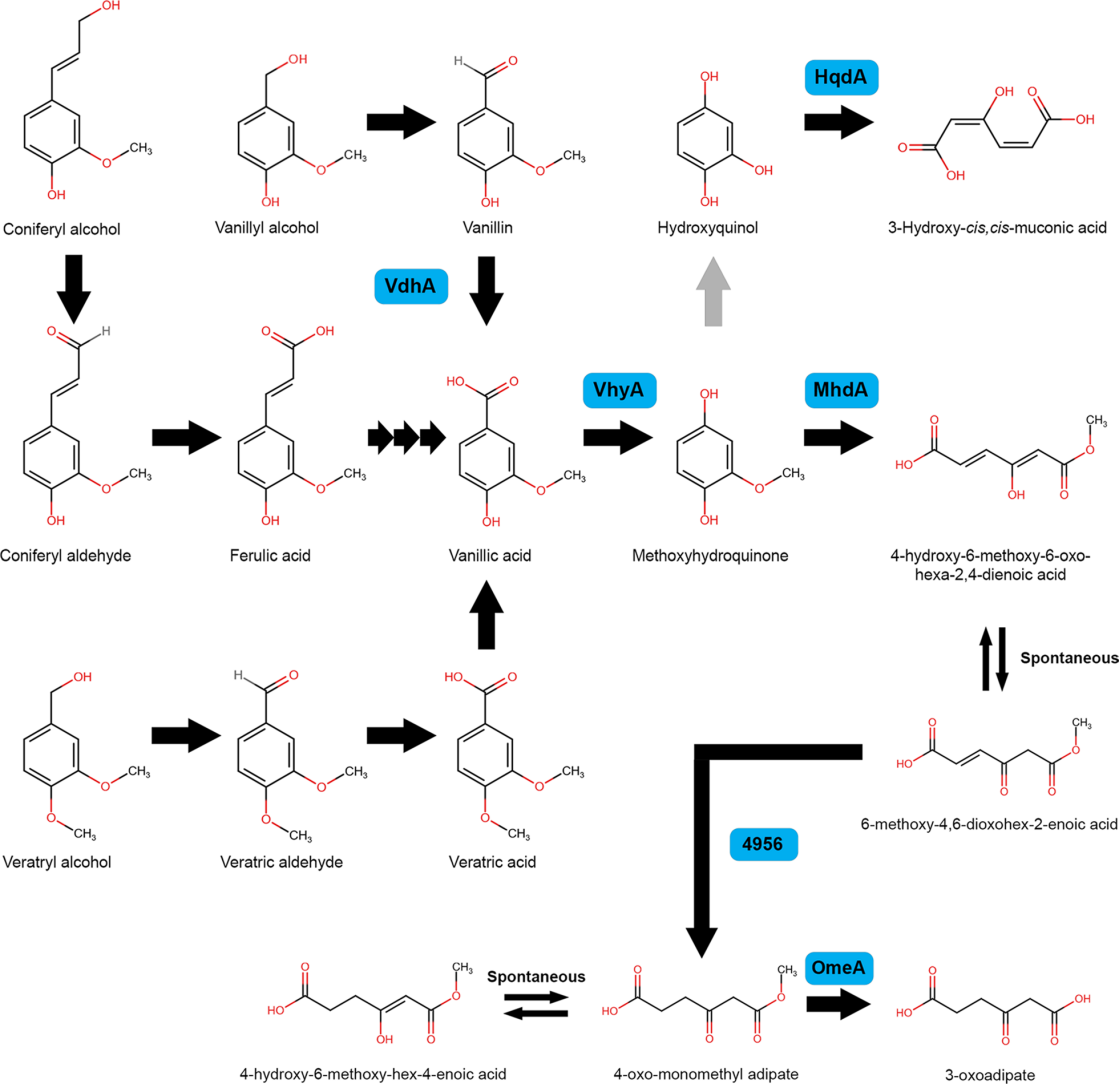
Overview of the ferulic acid and vanillic acid metabolic pathway and the suggested pathway after ring cleavage of methoxyhydroquinone in *A. niger*. Arrows in black are confirmed conversions and in grey are suggested conversions. Multiple arrows indicate conversions with multiple steps

Phylogenetic analysis of VhyA and MhdA revealed that these enzymes are conserved in *A. oryzae, Aspergillus flavus*, *Aspergillus terreus*, *A. fumigatus* and *A. nidulans*, which were all able to convert vanillic acid to methoxyhydroquinone [15]. No homologs of *Trichoderma* species clustered with VhyA or MhdA, which correlates with the observation that *Trichoderma* converts vanillic acid to vanillin and vanillyl alcohol and not to methoxyhydroquinone. In *A. nidulans,* a homogentisate 1,2-dioxygenase (HmgA, AN1897) has been characterized [44]. However, BLASTP with the amino acid sequence of HmgA revealed that it shares only 50.8% identity with MhdA while NRRL3_9969 is 87% similar to HmgA. Alternative pathways were suggested in several *Aspergillus* species, in which vanillic acid was converted to protocatechuic acid or guaiacol [45], but these were not observed in *A. niger* [15]. Our study supports this observation since protocatechuic acid or guaiacol were not detected during the accumulation experiment with Δ*vhyA* or Δ*mhdA* on ferulic acid, vanillic acid and vanillin. Next to that, we previously showed that deletion of protocatechuic acid 3,4-dioxygenase (*prcA*) and/or hydroxyquinol 1,2-dioxygenase (*hqdA*, NRRL3_2644) does not result in reduced growth on ferulic acid, vanillin or vanillic acid [39]. Interestingly, *hqdA* was upregulated by coniferyl alcohol, ferulic acid and vanillic acid (Additional file 2: Table S2). However, deletion of *hqdA* did not result in a phenotype on ferulic acid nor did HqdA show activity on methoxyhydroquinone [39]. This suggests that methoxyhydroquinone can be converted to hydroxyquinol, which was also proposed for *Paecilomyces variotii* and *S. pulverulentum* [35, 46]. However, the deletion of *mhdA* resulted in severely reduced growth on vanillin and vanillic acid and indicates that the conversion of methoxyhydroquinone to hydroxyquinol plays a minor role. The presence of this pathway could explain why deletion of *mhdA* does not result in complete accumulation of methoxyhydroquinone when grown on coniferyl alcohol, ferulic acid or vanillic acid. Based on previous observations and our transcriptome data, phenotypic profile and enzymatic assays, we suggest an updated version of the vanillic acid metabolic pathway of *A. niger* (Fig. 6).

Deletion of *omeA* resulted in the accumulation of 4-oxo-monomethyl adipate (6-methoxy-4,6-dioxohexanoic acid), which is a reduction product of 4-hydroxy-6-methoxy-6-oxohexa-2,4-dienoic acid. NRRL3_10110 is annotated as an esterase and therefore it is likely that it converts 4-oxo-monomethyl adipate to 3-oxoadipate, which is a commonly observed aromatic ring cleavage product [11]. This means that this gene encodes for a 4-oxo-monomethyl adipate esterase (OmeA). We included this in the suggested pathway (Fig. 6). After 4-hydroxy-6-methoxy-6-oxohexa-2,4-enedioic acid is formed, it is most likely converted spontaneously through tautomerization to 6-methoxy-4,6-dioxohexa-2-enoic acid. Then, 4-oxo-monomethyl adipate is converted to 6-methoxy-4,6-dioxohexanoic acid catalyzed by a reductase. This type of reaction is also observed in the degradation of hydroxyquinol, in which maleylacetate is reduced to 3-oxoadipate [47–50]. Using the whole-genome transcriptome dataset, a candidate reductase (NRRL3_4956) (Additional file 2: Table S2), which shared homology with maleylacetate reductase of *Pseudomonas* sp. (Uniprot: P27101) [50], was identified and is likely to be involved in the reduction of 6-methoxy-4,6-dioxohexa-2-enoic acid. 4-oxo-monomethyl adipate is finally converted to 3-oxoadipate by OmeA (Fig. 6). Rapid, spontaneous tautomerization was directly observed for 4-oxo-monomethyl adipate during ^1^H-NMR analysis, by the exchange of the C5 protons with deuterium via the enolic form (Additional file 1: Fig. S5); similarly, equilibration between 4-hydroxy-6-methoxy-6-oxohexa-2,4-enedioic acid and 6-methoxy-4,6-dioxohex-2-enoic acid occurs readily in aqueous solution, without enzymatic assistance.

In the *A. niger* genome sequence, the gene encoding for OmeA neighbors with *mhdA* and NRRL3_10109, annotated as an MFS-type tetracycline resistance protein, which was also upregulated by coniferyl alcohol, ferulic acid, vanillic acid and veratric acid (Additional file 2: Table S2). However, the involvement of this gene in the degradation of aromatic compounds remains to be studied.

The knowledge about the conversion of ferulic acid, vanillic acid and vanillin is important for new biotransformation strategies in fungi, for which better understanding of the aromatic metabolic pathways and the involved genes and enzymes involved is crucial. The identification and characterization of the novel *A. niger* enzymes VdhA, VhyA, MhdA and OmeA contributes greatly to a better understanding of these fungal aromatic metabolic pathways and revealed a novel metabolic pathway for the degradation of methoxyhydroquinone. In addition, we demonstrated that this knowledge can be applied to create fungal cell factories that can produce aromatic compounds such as vanillic acid and methoxyhydroquinone.

## Materials and methods

### Growth conditions

*Aspergillus niger* strains used in this study are shown in Table 2. The fungi were grown on complete medium (CM) [51] agar (1.5% w/v) plates at 30 °C for 4 days. Spores were harvested with 10 mL *N-*(2-acetamido)-2-aminoethanesulfonic acid buffer. Minimal medium (MM) [51] agar (1.5% w/v) plates were inoculated with 10^3^ freshly isolated spores. MM plates for growth profile experiments were supplemented with aromatic compounds as sole carbon source. Due to the toxicity of the aromatic compounds different concentrations were used for the growth profile, i.e. 2 mmol/L for benzoic acid, ferulic acid and vanillin, while 5 mmol/L was used for the remaining aromatic compounds. All aromatic compounds and chemicals were purchased from Sigma Aldrich.

**Table 2:** Strains used in this study

Strains	CBS number	Genotype	References
N402	CBS 141247	*cspA1*	[56]
N593 Δ*kusA*	CBS 138852	*cspA1, pyrA,* Δ*kusA::amdS*	[57]
Reference	CBS 145984	*cspA1, pyrA,* Δ*kusA::amdS,* Δ*pyrA::pyrG*	[54]
Δ*vdhA*	CBS 145978	*cspA1, pyrA,* Δ*kusA::amdS,* Δ*vdhA::pyrG*	This study
Δ*vhyA*	CBS 145979	*cspA1, pyrA,* Δ*kusA::amdS,* Δ*vhyA::pyrG*	This study
Δ*mhdA*	CBS 145981	*cspA1, pyrA,* Δ*kusA::amdS,* Δ*mhdA::pyrG*	This study
Δ*omeA*	CBS 145980	*cspA1, pyrA,* Δ*kusA::amdS,* Δ*omeA::pyrG*	This study

Transfer experiments were performed as described previously [39]. Equal portions of mycelia were transferred to flasks containing 50 mL MM and 0.02% (w/v) coniferyl alcohol, vanillic acid or veratric acid. The cultures were incubated on a rotary shaker for 2 h at 30 °C, 250 rpm. Mycelia were harvested, dried between tissue paper to remove excess liquid and frozen in liquid nitrogen.

### RNA extraction and RNA sequencing

RNA was extracted as described previously [39]. The quality and quantity of RNA were determined by gel electrophoresis and RNA6000 Nano Assay using the Agilent 2100 Bioanalyzer (Agilent Technologies, Santa Clara, CA, USA). RNA sequencing was conducted by BGI Tech Solutions (Tai Po, Hong Kong) using the Illumina HiseqTM 2000 platform (Illumina Inc., San Diego, CA, USA). Transcriptome data of *A. niger* N402 grown on ferulic acid and no carbon source was obtained from GEO Accession number GSE135001 [25] and GSE13499 [39], respectively. Transcriptome analysis was performed as previously described [52]. The transcriptome data was stored at the NCBI Gene expression omnibus, under the GEO Accession number GSE154865.

### Protoplast-mediated transformation of *A. niger*

All deletion strains were made through protoplast-mediated transformation of *A. niger* N593 Δ*kusA* by homologous recombination using deletion cassettes containing 900–1000 bp upstream and downstream of the target gene fused to the orotidine 5ʹ-phosphate decarboxylase (*pyrG*) gene from *A. oryzae* RIB40. The used primers are listed in Additional file 2: Table S3. The protoplast-mediated transformation and purification of the selected transformants was performed as described previously [52]. Transformants were verified with diagnostic PCR using primers listed in Additional file 2: Table S3.

### Accumulation of aromatic compounds

Transfer experiments were conducted as indicated above. Equal portions of mycelia were transferred to 250 mL flasks containing 50 mL MM and 5 mmol/L coniferyl alcohol, ferulic acid, vanillyl alcohol, vanillin, vanillic acid, veratryl alcohol, veratric aldehyde or veratric acid. The cultures were incubated on a rotary shaker for 24 h at 30 °C, 250 rpm. Supernatant samples were collected after 24 h and diluted 50 times with acetonitrile before HPLC analysis using the setup described previously [53].

### Production of recombinant VdhA, VhyA and MhdA

Full-length *vdhA*, *vhyA* and *mhdA* were synthesized based on their reference sequence (NRRL3_3887, NRRL3_9897 and NRRL3_10111, respectively) in pET23b containing a C-terminal hexa his-tag (Genscript Biotech, Leiden, the Netherlands) and used to transform the *E. coli* protein production strain BL-21 DE3 (New England Biolabs, Ipswich, MA). *E. coli* BL-21 DE3 pET23a-*vdhA*, pET23a-*vhyA* and pET23a-*mhdA* were grown in LB medium supplemented with 50 µg/mL ampicillin at 37 °C, 160 rpm, until an OD600 of 0.6–0.8 was reached. At this time, 100 µmol/L IPTG was added to the cultures, which were then further incubated for 24 h at 12 °C, 160 rpm. The cultures were subsequently centrifuged at 3.2×*g*, 4 °C for 10 min. Pellets were dissolved in 5 mL (per 100 mL culture) BugBuster Protein Extraction Reagent (Novagen), containing 1 KU Lysozyme/mL (Sigma-Aldrich), 25 U Benzonase^®^ Nuclease and cOmplete™, EDTA-free Protease Inhibitor Cocktail and incubated for 20 min at 4 °C, with gentle rocking. The cell debris of each sample was removed by centrifugation at 4 °C and the supernatant containing the soluble fraction of proteins was isolated. For enzyme purification we used the HisTrap FF 1 mL column coupled with the ÄKTA start system (GE Healthcare Life Sciences, Uppsala, Sweden) as described previously [39]. After purification, a final concentration of 0.5 mmol/L FAD was added to VhyA.

### Enzyme assay of VdhA, VhyA and MhdA

Purified enzymes were used for the assays. The reaction mixture for VdhA contained McIlvaine buffer, pH 7.0, consisting of 0.1 mol/L citric acid and 0.2 mol/L phosphate buffer, 500 µmol/L vanillin, 500 µmol/L NAD^+^ and 5 µL purified VdhA. The reaction mixture for VhyA contained McIlvaine buffer, pH 7.0, consisting of 0.1 mol/L citric acid and 0.2 mol/L phosphate buffer, 500 µmol/L vanillic acid, 500 µmol/L NADH and 5 µL purified VhyA. The reaction mixture for MhdA contained 0.2 mol/L HEPES buffer, pH 6.0, 500 µmol/L methoxyhydroquinone, 50 µmol/L FeSO_4_ and 5 µL purified MhdA. All reactions were incubated at 30 °C for 1 h and stopped by heating at 80 °C for ten minutes. The samples were diluted ten times and analyzed using HPLC.

### Identification of 4-hydroxy-6-methoxy-6-oxohexa-2,4-dienoic acid

The MhdA reaction mixture, containing 0.1 mol/L phosphate buffer, pH 6.0, 250 µmol/L methoxyhydroquinone, 50 µmol/L FeSO_4_ and 5 µL purified MhdA with a total volume of 5 mL, was analyzed with HPLC–MS before and after extraction with ethyl acetate. The extraction was performed with 10 mL reaction mixture by adjusting the pH to pH 2 and the water-soluble analytes were salted out with brine to ethyl acetate. The extraction was repeated three times and finally the product mixture was collected by evaporation of the solvent under vacuum in a rotary evaporator. The sample (5 mg) was dissolved in 1% formic acid in 1:10 acetonitrile:H_2_O as 1 mg/mL for high resolution LC–HRMS analysis.

The LC–HRMS analysis was performed on Thermo Scientific Orbitrap Fusion mass spectrometer (San Jose, USA) connected to Thermo Scientific Dionex Ultimate 3000 ultrahigh performance liquid chromatograph (Germering, Germany). The sample injection volume was 5 μL. LC separation was done using Phenomenex Luna^Ⓡ^ Omega Polar C18 (1.6 µm, 100 × 2.1 mm) column at 40 °C using a non-linear gradient (curve 7) of two mobile phases: 0.1% formic acid in water (A) and 0.1% formic acid in acetonitrile (B). The gradient with flow rate of 0.3 mL/min was run from 1% B at 0 min to 95% B at 15 min. After this the B eluent was kept 95% for 1 min, returned to 5% during 1 min and equilibrated at 5% for 3 min.

The ionization was done using thermal heated electrospray HESI probe both in the positive and negative ion mode. The instrumental parameters were set as follows: positive ion spray voltage 3500 V, negative ion spray voltage 2500 V, source temperature 300 °C, ion transfer tube temperature 350 °C, sheath gas 40, auxiliary gas 15 and sweep gas 0. Mass measurement was done with mass range *m*/*z* 70–350 using RF lens at 60% and quadrupole isolation (*m*/*z* 70–350) at resolution of 120,000. Mass accuracy of the instrument using external calibration for both positive and negative ion mode was specified to be ≤ 3 ppm.

### Identification of 6-methoxy-4,6-dioxohexanoic acid

For the identification of 6-methoxy-4,6-dioxohexanoic acid, the accumulated product of ∆*omeA* grown on vanillic acid was isolated through ethyl acetate extraction. The organic phase was dried, dissolved in acetonitrile and analyzed for the presence of 6-methoxy-4,6-dioxohexanoic acid by HPLC. For NMR analysis, the aqueous sample was washed three times with CDCl_3_. The aqueous layer was concentrated to dryness *in vacuo* (50 °C) and redissolved in D_2_O. The ^1^H-NMR spectrum was recorded on an Agilent MRF 400 equipped with a OneNMR probe and Optima Tune system. The signals of the major species correspond to 6-methoxy-4,6-dioxohexanoic acid (bisdeuterated at C5). The recorded spectrum is in good agreement with spectral data reported in the literature for the proposed structure [29].

### Phylogenetic analysis

The amino acid sequences of VhyA and MhdA were used for BLASTP analyses on selected ascomycete and basidiomycete genomes as described previously (Additional file 2: Table S4) [54]. To reduce the amount of insignificant hits, a cutoff E-value of e−40 was used. Several amino acid sequences were curated manually or with the gene prediction software, Augustus (Additional file 2: Table S5) [55]. The bacterial enzymes, Vdh (Uniprot, O05619) from *Pseudomonas* sp. strain HR199 and LigV (Uniprot, A2PZP3) from *Sphingomonas paucimobilis* were added manually to the multiple alignment. The Maximum Likelihood, Neighbor Joining and Minimum Evolution trees were constructed using MEGA 7 with 500 bootstraps and complete deletion of gaps.

## Supplementary Information

**Additional file 1: Fig S1.** Visualization of VdhA, VhyA and MhdA by SDS-PAGE. **Fig. S2.** Conversion of aromatic compounds by the reference (a), ∆*vdhA* (b), ∆*vhyA* (c)*,* and ∆*mhdA* (d) after 24 h of incubation**.** Concentrations of the detected compounds can be found in Table 1. Error bars represent the standard deviation between three biological replicates. **Fig. S3.** Maximum likelihood (ML; 500 bootstraps) phylogenetic tree of *A. niger* VdhA compared to selected fungal genomes. The scale bar shows a distance equivalent to 0.2 amino acid substitutions per site. Values over 50% bootstrap support are shown with ML values in black, Neighbor Joining values in purple and Minimum Evolution values in blue. Characterized enzymes are in bold. Blue font represents ascomycete fungi, red font basidiomycete fungi, green font bacteria, and orange font Saccharomycetes. Fungal species names are followed by protein IDs from JGI (http://genome.jgi-psf.org/programs/fungi/index.jsf). **Fig. S4.** Maximum likelihood (ML; 500 bootstraps) phylogenetic tree of *A. niger* MhdA compared to selected fungal genomes. The scale bar shows a distance equivalent to 0.2 amino acid substitutions per site. Values over 50% bootstrap support are shown with ML values in black, Neighbor Joining values in purple and Minimum Evolution values in blue. In bold are characterized enzymes. Blue font represents ascomycete fungi, red font basidiomycete fungi, green font bacteria, black font plants and pink font *Homo sapiens*. Fungal species names are followed by protein IDs from JGI (http://genome.jgi-psf.org/programs/fungi/index.jsf). **Fig. S5.** ^1^H-NMR spectrum of 4-oxo-monomethyl adipate (D_2_O). The structure corresponds to 4-oxo-monomethyl adipate with D^2^ at C5 position.

**Additional file 2: Table S1.** Molar and mass yield on vanillin, vanillic acid or methoxyhydroquinone production from 5 mmol/L on guaiacyl units and related aromatic compounds obtained by ∆*vdhA*, ∆*vhyA* and ∆*mhdA*. Mean values and standard deviations of three biological replicates. **Table S2.** Transcriptome data of *hqdA* and NRRL3_4956. Fold change (log2) compared to a no carbon source control and *p-*values were calculated with DESeq2. **Table S3.** Primers used in this study. In red are the regions overlapping *pyrG*. **Table S4.** Fungal genomes used for the phylogenetic study of VdhA and MhdA. Genomes were obtained from JGI MycoCosm (https://mycocosm.jgi.doe.gov/mycocosm/home). **Table S5.** Amino acid sequences used for the phylogenetic analysis of MhdA and VdhA that were curated manually or with the gene prediction software Augustus.

## Data Availability

The datasets generated and/or analyzed during the current study are available in the NCBI Gene expression omnibus repository, under the GEO Accession number GSE154865.

## Abbreviations

MhdA: Methoxyhydroquinone 1,2-dioxygenase
OmeA: 4-Oxo-monomethyl adipate esterase
VhyA: Vanillic acid hydroxylase
VdhA: Vanillin dehydrogenase

## References

[1] Banerjee G, Chattopadhyay P (2019). Vanillin biotechnology: the perspectives and future. J Sci Food Agric.

[2] Galadima AI, Salleh MM, Hussin H, Chong CS, Yahya A, Mohamad SE, Abd-Aziz S, Yusof NNM, Naser MA, Al-Junid AFM (2020). Biovanillin: production concepts and prevention of side product formation. Biomass Convers Biorefin.

[3] Ali WI, Al-Abbasy OY, Younis SA (2021). Vanillic acid: an antioxidant used in preventing browning process in pear (pyruscommunisl.) juice. Ann Rom Soc Cell Biol.

[4] Amarasekara AS, Wiredu B, Razzaq A (2012). Vanillin based polymers: I. An electrochemical route to polyvanillin. Green Chem.

[5] Nikafshar S, Zabihi O, Hamidi S, Moradi Y, Barzegar S, Ahmadi M, Naebe M (2017). A renewable bio-based epoxy resin with improved mechanical performance that can compete with DGEBA. RSC Adv.

[6] Chu F, Ma C, Zhang T, Xu Z, Mu X, Cai W, Zhou X, Ma S, Zhou Y, Hu W, Song L (2020). Renewable vanillin-based flame retardant toughening agent with ultra-low phosphorus loading for the fabrication of high-performance epoxy thermoset. Compos Part B Eng.

[7] Fache M, Viola A, Auvergne R, Boutevin B, Caillol S (2015). Biobased epoxy thermosets from vanillin-derived oligomers. Eur Polym J.

[8] Bassett AW, Honnig AE, Breyta CM, Dunn IC, La Scala JJ, Stanzione JF (2020). Vanillin-based resin for additive manufacturing. ACS Sustain Chem Eng.

[9] Kaur B, Chakraborty D (2013). Biotechnological and molecular approaches for vanillin production: a review. Appl Biochem Biotechnol.

[10] Lesage-Meessen L, Delattre M, Haon M, Thibault JF, Ceccaldi BC, Brunerie P, Asther M (1996). A two-step bioconversion process for vanillin production from ferulic acid combining *Aspergillus niger* and *Pycnoporus cinnabarinus*. J Biotechnol.

[11] Lubbers RJM, Dilokpimol A, Visser J, Mäkelä MR, Hildén KS, de Vries RP (2019). A comparison between the homocyclic aromatic metabolic pathways from plant-derived compounds by bacteria and fungi. Biotechnol Adv.

[12] Fitzgerald DJ, Stratford M, Narbad A (2003). Analysis of the inhibition of food spoilage yeasts by vanillin. Int J Food Microbiol.

[13] Fitzgerald DJ, Stratford M, Gasson MJ, Ueckert J, Bos A, Narbad A (2004). Mode of antimicrobial of vanillin against *Escherichia coli*, *Lactobacillus plantarum* and *Listeria innocua*. J Appl Microbiol.

[14] Chow KT, Pope MK, Davies J (1999). Characterization of a vanillic acid non-oxidative decarboxylation gene cluster from *Streptomyces* sp. D7. Microbiology.

[15] Guiraud P, Steiman R, Seigle-Murandi F, Benoit-Guyod JL (1992). Metabolism of vanillic acid by Micromycetes. World J Microbiol Biotechnol.

[16] Abe T, Masai E, Miyauchi K, Katayama Y, Fukuda M (2005). A tetrahydrofolate-dependent *O*-demethylase, LigM, is crucial for catabolism of vanillate and syringate in *Sphingomonas paucimobilis* SYK-6. J Bacteriol.

[17] El-Mansi EMT, Anderson SCK (2004). The hydroxylation of vanillate and its conversion to methoxyhydroquinone by a strain of *Pseudomonas fluorescens* devoid of demethylase and methylhydroxylase activities. World J Microbiol Biotechnol.

[18] Gallage NJ, Møller BL (2015). Vanillin-bioconversion and bioengineering of the most popular plant flavor and its de novo biosynthesis in the vanilla orchid. Mol Plant.

[19] Zheng L, Zheng P, Sun Z, Bai Y, Wang J, Guo X (2007). Production of vanillin from waste residue of rice bran oil by *Aspergillus niger* and *Pycnoporus cinnabarinus*. Bioresour Technol.

[20] Lesage-Meessen L, Stentelaire C, Lomascolo A, Couteau D, Asther M, Moukha S, Record E, Sigoillot JC, Asther M (1999). Fungal transformation of ferulic acid from sugar beet pulp to natural vanillin. J Sci Food Agric.

[21] Busswell JA, Eriksson K-E, Petterson B (1981). Purification and partial characterization of vanillate hydroxylase(decarboxylating) from *Sporotrichum pulverulentum*. J Chromatogr.

[22] Busswell JA, Eriksson K-E (1988). Vanillate hydroxylase from *Sporotrichum pulverulentum*. Methods Enzymol.

[23] Yajima Y, Enoki A, Mayfield MB, Gold MH (1979). Vanillate hydroxylase from the white rot basidiomycete *Phanerochaete chrysosporium*. Arch Microbiol.

[24] Baqueiro-Peña I, Rodríguez-Serrano G, González-Zamora E, Augur C, Loera O, Saucedo-Castañeda G (2010). Biotransformation of ferulic acid to 4-vinylguaiacol by a wild and a diploid strain of *Aspergillus niger*. Bioresour Technol.

[25] Lubbers RJM, Liwanag AJ, Peng M, Dilokpimol A, Benoit-Gelber I, de Vries RP (2020). Evolutionary adaptation of *Aspergillus niger* for increased ferulic acid tolerance. J Appl Microbiol.

[26] Lubbers RJM, Dilokpimol A, Visser J, de Vries RP (2021). *Aspergillus niger* uses the peroxisomal CoA-dependent β-oxidative genes to degrade the hydroxycinnamic acids caffeic acid, ferulic acid, and *p*-coumaric acid. Appl Microbiol Biotechnol.

[27] de Jong E, Beuling EE, van der Zwan RP, de Bont JAM (1990). Degradation of veratryl alcohol by *Penicillium simplicissimum*. Appl Microbiol Biotechnol.

[28] Priefert H, Rabenhorst J, Steinbuchel A, Rabenhorst R (1997). Molecular characterization of genes of *Pseudomonas* sp. strain HR199 involved in bioconversion of vanillin to protocatechuate. J Bacteriol.

[29] Oku A, Numata M (2000). Three- to six-carbon ring-enlargement reaction of cyclic ortho esters bearing a diazocarbonyl side chain. Use of the intramolecular formation of tricyclooxonium ylides. J Org Chem.

[30] Lubbers RJM, de Vries RP (2021). Production of protocatechuic acid from *p*-hydroxyphenyl (H) units and related aromatic compounds using an *Aspergillus niger* cell factory. MBio.

[31] Guiraud P, Steiman R, Seigle-murandi F, Benoit-guyod JL (1995). Comparison of the toxicity of various lignin-related phenolic compounds toward selected fungi perfecti and fungi imperfecti. Ecotoxicol Environ Saf.

[32] Gupta JK, Hamp SG, Buswell JA, Eriksson KE (1981). Metabolism of trans-ferulic acid by the white-rot fungus *Sporotrichum pulverulentum*. Arch Microbiol.

[33] Kirk TK, Lorenz LF (1973). Methoxyhydroquinone, an intermediate of vanillate catabolism by *Polyporus dichrous*. Appl Microbiol.

[34] Nishida A, Fukuzumi T (1978). Formation of coniferyl alcohol from ferulic acid by the white rot fungus *Trametes*. Phytochemistry.

[35] Rahouti M, Seigle-Murandi F, Steiman R, Eriksson KE (1989). Metabolism of ferulic acid by *Paecilomyces variotii* and *Pestalotia palmarum*. Appl Environ Microbiol.

[36] Falconnier B, Lapierre C, Lesage-Meessen L, Yonnet G, Brunerie P, Colonna-Ceccaldi B, Corrieu G, Asther M (1994). Vanillin as a product of ferulic acid biotransformation by the white-rot fungus *Pycnoporus cinnabarinus* I-937: identification of metabolic pathways. J Biotechnol.

[37] Arentshorst M, Di FM, Moisan M, Reid ID, Spaapen TOM, Van DJ, Demirci E, Powlowski J (2021). Identification of a conserved transcriptional activator-repressor module controlling the expression of genes involved in tannic acid degradation and gallic acid utilization in *Aspergillus niger*. Front Fungal Biol.

[38] Westphal AH, Tischler D, van Berkel WJH (2021). Natural diversity of FAD-dependent 4-hydroxybenzoate hydroxylases. Arch Biochem Biophys.

[39] Lubbers RJM, Dilokpimol A, Peng M, Visser J, Mäkelä MR, Hildén KS, de Vries RP (2019). Discovery of novel *p*-hydroxybenzoate-*m*-hydroxylase, protocatechuate 3,4 ring-cleavage dioxygenase, and hydroxyquinol 1,2 ring-cleavage dioxygenase from the filamentous fungus *Aspergillus niger*. ACS Sustain Chem Eng.

[40] Ding W, Si M, Zhang W, Zhang Y, Chen C, Zhang L, Lu Z, Chen S, Shen X (2015). Functional characterization of a vanillin dehydrogenase in *Corynebacterium glutamicum*. Sci Rep.

[41] Masai E, Yamamoto Y, Inque T, Takamura K, Hara H, Kasai D, Katayama Y, Fukuda M (2007). Characterization of LigV essential for catabolism of vanillin by *Sphingomonas paucimobilis* SYK-6. Biosci Biotechnol Biochem.

[42] Graf N, Wenzel M, Altenbuchner J (2016). Identification and characterization of the vanillin dehydrogenase YfmT in *Bacillus subtilis* 3NA. Appl Microbiol Biotechnol.

[43] Kim DS, Robertson GP, Guiver MD (2008). Comb-shaped poly(arylene ether sulfone)s as proton exchange membranes. Macromolecules.

[44] Fernandez-Canon JM, Penalva MA (1995). Molecular characterization of a gene encoding a homogentisate dioxygenase from *Aspergillus nidulans* and identification of its human and plant homologues. J Biol Chem.

[45] Milstein O, Vered Y, Shragina L, Gressel J, Flowers HM, Hüttermann A (1983). Metabolism of lignin related aromatic compounds by *Aspergillus japonicus*. Arch Microbiol.

[46] Ander P, Hatakka A, Eriksson KE (1980). Vanillic acid metabolism by the white-rot fungus *Sporotrichum pulverulentum*. Arch Microbiol.

[47] Travkin VM, Solyanikova IP, Golovleva LA (2006). Hydroxyquinol pathway for microbial degradation of halogenated aromatic compounds. J Environ Sci Health Part B Pestic Food Contam Agric Wastes.

[48] Spence EM, Scott HT, Dumond L, Calvo-Bado L, di Monaco S, Williamson JJ, Persinoti GF, Squina FM, Bugg TDH (2020). Characterisation of the hydroxyquinol degradation pathway in *Rhodococcus jostii* RHA1 and *Agrobacterium* sp.: an alternative pathway for degradation of protocatechuic acid and lignin degradation fragments. Appl Environ Microbiol.

[49] Holesova Z, Jakubkova M, Zavadiakova I, Zeman I, Tomaska L, Nosek J (2011). Gentisate and 3-oxoadipate pathways in the yeast *Candida parapsilosis*: identification and functional analysis of the genes coding for 3-hydroxybenzoate 6-hydroxylase and 4-hydroxybenzoate 1-hydroxylase. Microbiology.

[50] van der Meer JR, Eggen RIL, Zehnder AJB, de Vos WM (1991). Sequence analysis of the *Pseudomonas* sp. strain P51 tcb gene cluster, which encodes metabolism of chlorinated catechols: evidence for specialization of catechol 1,2-dioxygenases for chlorinated substrates. J Bacteriol.

[51] de Vries RP, Frisvad JC, van de Vondervoort PJI, Burgers K, Kuijpers AFA, Samson RA, Visser J (2005). *Aspergillus vadensis*, a new species of the group of black Aspergilli. Antonie van Leeuwenhoek Int J Gen Mol Microbiol.

[52] Kowalczyk JE, Lubbers RJM, Peng M, Battaglia E, Visser J, de Vries RP (2017). Combinatorial control of gene expression in *Aspergillus niger* grown on sugar beet pectin. Sci Rep.

[53] Dilokpimol A, Mäkelä MR, Mansouri S, Belova O, Waterstraat M, Bunzel M, de Vries RP, Hildén KS (2017). Expanding the feruloyl esterase gene family of *Aspergillus niger* by characterization of a feruloyl esterase, FaeC. N Biotechnol.

[54] Lubbers RJM, Dilokpimol A, Visser J, Hildén KS, Mäkelä MR, de Vries RP (2021). Discovery and functional analysis of a salicylic acid hydroxylase from *Aspergillus niger*. Appl Environ Microbiol.

[55] Stanke M, Steinkamp R, Waack S, Morgenstern B (2004). AUGUSTUS: a web server for gene finding in eukaryotes. Nucleic Acids Res.

[56] Bos CJ, Debets AJM, Swart K, Huybers A, Kobus G, Slakhorst SM (1988). Genetic analysis and the construction of master strains for assignment of genes to six linkage groups in *Aspergillus niger*. Curr Genet.

[57] Meyer V, Arentshorst M, El-Ghezal A, Drews AC, Kooistra R, van den Hondel CAMJJ, Ram AFJ (2007). Highly efficient gene targeting in the *Aspergillus niger* kusA mutant. J Biotechnol.

