# Supplementary material for: Vanillic acid and methoxyhydroquinone production from guaiacyl units and related aromatic compounds using Aspergillus niger cell factories
Source: Microb Cell Fact. 2021 Aug 3;20:151. doi: 10.1186/s12934-021-01643-x (PMC8336404; doi:10.1186/s12934-021-01643-x)
Supplement: Supplementary file 1 — Additional file 1: Fig S1. Visualization of VdhA, VhyA and MhdA by SDS-PAGE. Fig. S2. Conversion of aromatic compounds by the reference (a), ∆vdhA (b), ∆vhyA (c), and ∆mhdA (d) after 24 h of incubation. Concentrations of the detected compounds can be found in Table 1. Error bars represent the standard deviation between three biological replicates. Fig. S3. Maximum likelihood (ML; 500 bootstraps) phylogenetic tree of A. niger VdhA compared to selected fungal genomes. The scale bar shows a distance equivalent to 0.2 amino acid substitutions per site. Values over 50% bootstrap support are shown with ML values in black, Neighbor Joining values in purple and Minimum Evolution values in blue. Characterized enzymes are in bold. Blue font represents ascomycete fungi, red font basidiomycete fungi, green font bacteria, and orange font Saccharomycetes. Fungal species names are followed by protein IDs from JGI (http://genome.jgi-psf.org/programs/fungi/index.jsf). Fig. S4. Maximum likelihood (ML; 500 bootstraps) phylogenetic tree of A. niger MhdA compared to selected fungal genomes. The scale bar shows a distance equivalent to 0.2 amino acid substitutions per site. Values over 50% bootstrap support are shown with ML values in black, Neighbor Joining values in purple and Minimum Evolution values in blue. In bold are characterized enzymes. Blue font represents ascomycete fungi, red font basidiomycete fungi, green font bacteria, black font plants and pink font Homo sapiens. Fungal species names are followed by protein IDs from JGI (http://genome.jgi-psf.org/programs/fungi/index.jsf). Fig. S5. 1H-NMR spectrum of 4-oxo-monomethyl adipate (D2O). The structure corresponds to 4-oxo-monomethyl adipate with D2 at C5 position. [file 12934_2021_1643_MOESM1_ESM.pdf]

Vanillic acid and methoxyhydroquinone production from guaiacyl units and related aromatic compounds using *Aspergillus niger* cell factories

Ronnie J.M. Lubbers,[1] *Adiphol Dilokpimol,*[1] *Paula A. Nousiainen,*[2] *Răzvan C. Cioc,*[3] *Jaap Visser,*[1] *Pieter C. A. Bruijnincx,*[3] *and Ronald P. de Vries*[1,*]

[1] *Fungal Physiology, Westerdijk Fungal Biodiversity Institute & Fungal Molecular Physiology, Utrecht University, Uppsalalaan 8, Utrecht, 3584CT, The Netherlands.*

[2] *Department of Chemistry, University of Helsinki, FI-00014, P.O. Box 55, A. I. Virtasen Aukio 1, Helsinki, Finland.*

[3] *Organic Chemistry and Catalysis, Debye Institute for Nanomaterials Science, Utrecht University, Universiteitsweg 99, 3584 CG Utrecht, The Netherlands.*

Corresponding Author
* Email: r.devries@wi.knaw.nl

Additional file 1

6 pages containing 5 figures

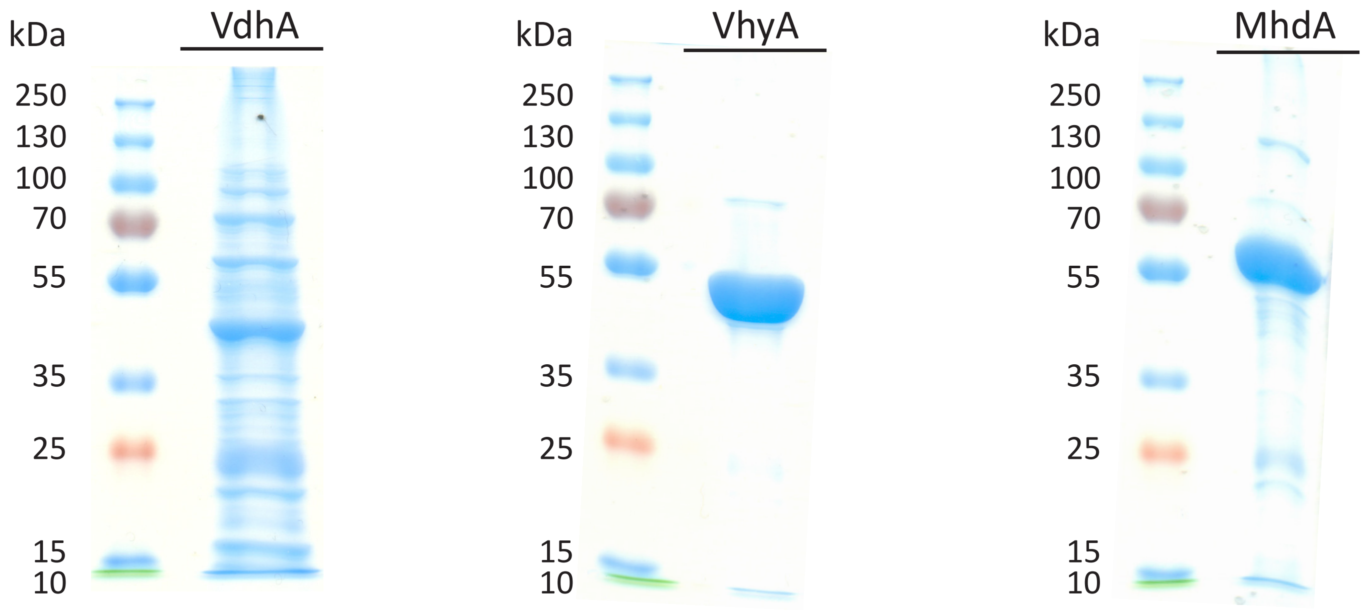

Fig S1. Visualization of VdhA, VhyA and MhdA by SDS-PAGE

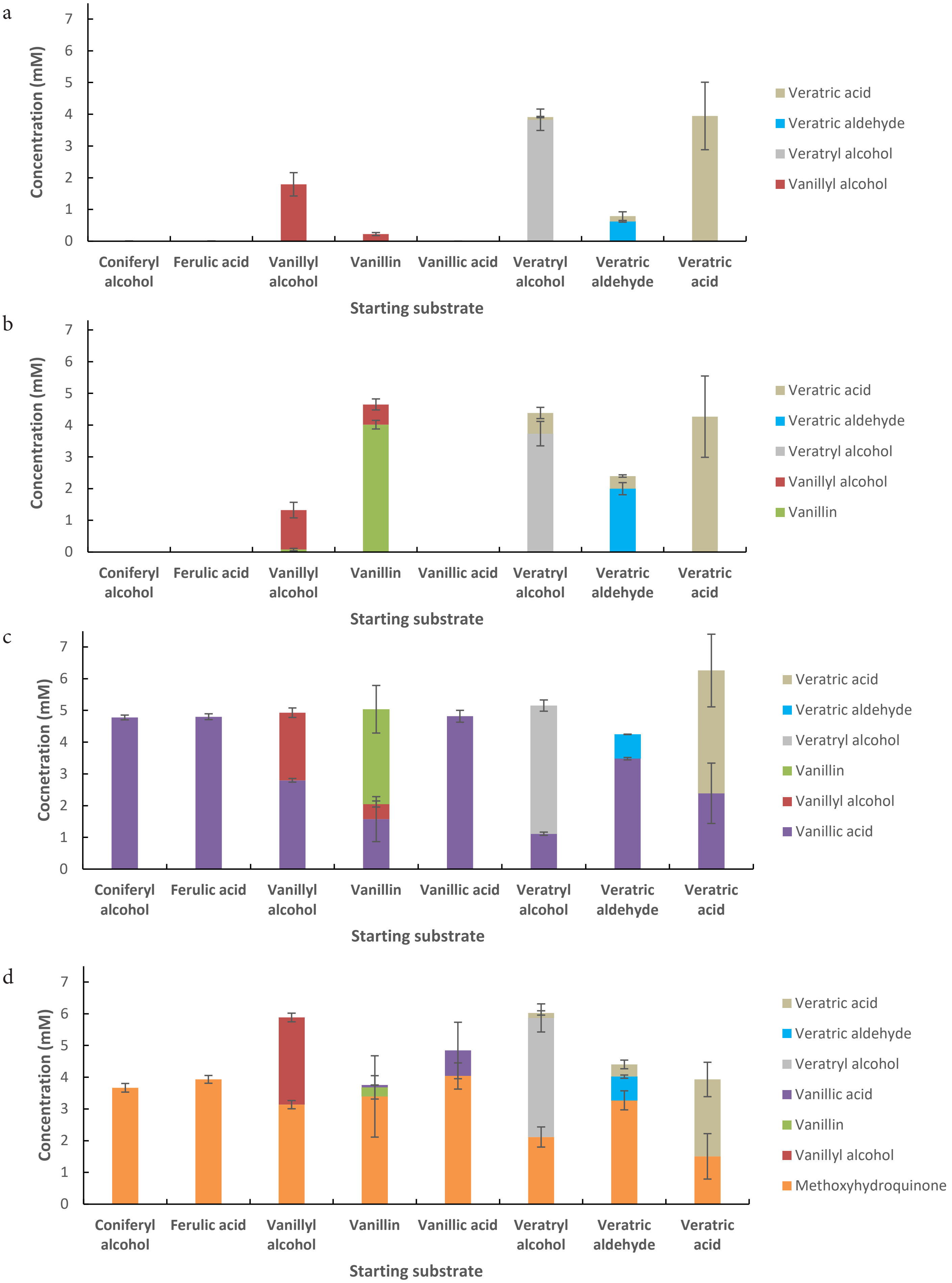

Fig. S2. Conversion of aromatic compounds by the reference (a), Δ*vdhA* (b), Δ*vhyA* (c), and Δ*mhdA* (d) after 24 hours of incubation. Concentrations of the detected compounds can be found in Table 1. Error bars represent the standard deviation between three biological replicates.

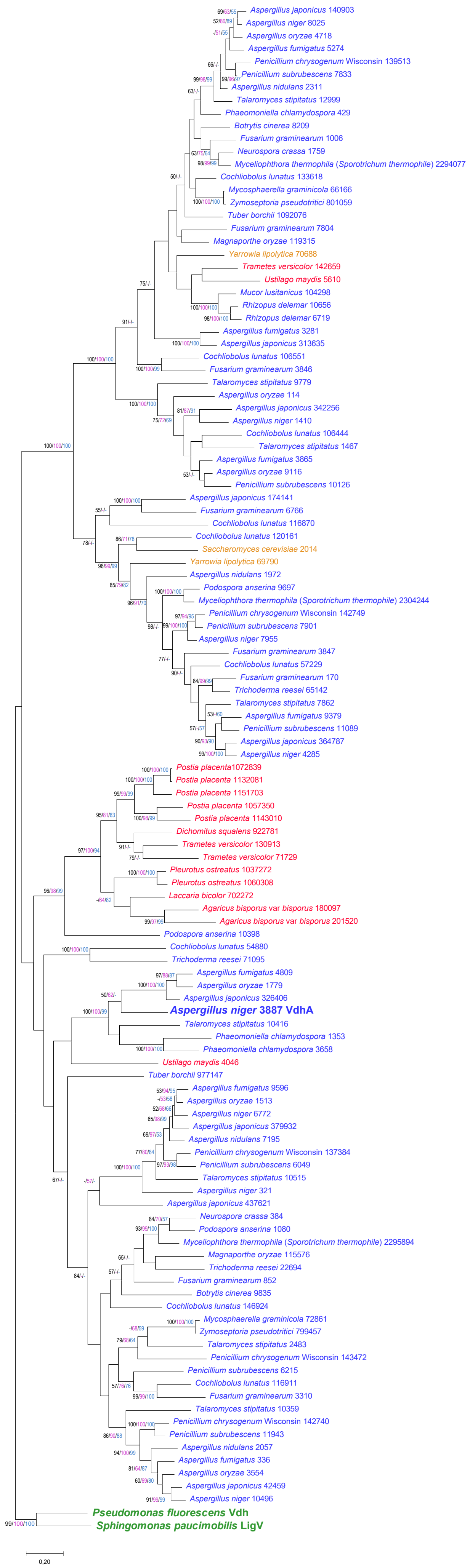
Aspergillus japonicus 140903
Aspergillus niger 8025
Aspergillus oryzae 4718
Aspergillus fumigatus 5274
Penicillium chrysogenum Wisconsin 139513
Penicillium subrubescens 7833
Aspergillus nidulans 2311
Talaromyces stipitatus 12999
Phaeomoniella chlamydospora 429
Botrytis cinerea 8209
Fusarium graminearum 1006
Neurospora crassa 1759
Myceliophthora thermophila (Sporotrichum thermophile) 2294077
Cochliobolus lunatus 133618
Mycosphaerella graminicola 66166
Zymoseptoria pseudotritici 801059
Tuber borchii 1092076
Fusarium graminearum 7804
Magnaporthe oryzae 119315
Yarrowia lipolytica 70688
Trametes versicolor 142659
Ustilago maydis 5610
Mucor lusitanicus 104298
Rhizopus delemar 10656
Rhizopus delemar 6719
Aspergillus fumigatus 3281
Aspergillus japonicus 313635
Cochliobolus lunatus 106551
Fusarium graminearum 3846
Talaromyces stipitatus 9779
Aspergillus oryzae 114
Aspergillus japonicus 342256
Aspergillus niger 1410
Cochliobolus lunatus 106444
Talaromyces stipitatus 1467
Aspergillus fumigatus 3865
Aspergillus oryzae 9116
Penicillium subrubescens 10126
Aspergillus japonicus 174141
Fusarium graminearum 6766
Cochliobolus lunatus 116870
Cochliobolus lunatus 120161
Saccharomyces cerevisiae 2014
Yarrowia lipolytica 69790
Aspergillus nidulans 1972
Podospora anserina 9697
Myceliophthora thermophila (Sporotrichum thermophile) 2304244
Penicillium chrysogenum Wisconsin 142749
Penicillium subrubescens 7901
Aspergillus niger 7955
Fusarium graminearum 3847
Cochliobolus lunatus 57229
Fusarium graminearum 170
Trichoderma reesei 65142
Talaromyces stipitatus 7862
Aspergillus fumigatus 9379
Penicillium subrubescens 11089
Aspergillus japonicus 364787
Aspergillus niger 4285
Postia placenta1072839
Postia placenta 1132081
Postia placenta 1151703
Postia placenta 1057350
Postia placenta 1143010
Dichomitus squalens 922781
Trametes versicolor 130913
Trametes versicolor 71729
Pleurotus ostreatus 1037272
Pleurotus ostreatus 1060308
Laccaria bicolor 702272
Agaricus bisporus var bisporus 180097
Agaricus bisporus var bisporus 201520
Podospora anserina 10398
Cochliobolus lunatus 54880
Trichoderma reesei 71095
Aspergillus fumigatus 4809
Aspergillus oryzae 1779
Aspergillus japonicus 326406
Aspergillus niger 3887 VdhA
Talaromyces stipitatus 10416
Phaeomoniella chlamydospora 1353
Phaeomoniella chlamydospora 3658
Ustilago maydis 4046
Tuber borchii 977147
Aspergillus fumigatus 9596
Aspergillus oryzae 1513
Aspergillus niger 6772
Aspergillus japonicus 379932
Aspergillus nidulans 7195
Penicillium chrysogenum Wisconsin 137384
Penicillium subrubescens 6049
Talaromyces stipitatus 10515
Aspergillus niger 321
Aspergillus japonicus 437621
Neurospora crassa 384
Podospora anserina 1080
Myceliophthora thermophila (Sporotrichum thermophile) 2295894
Magnaporthe oryzae 115576
Trichoderma reesei 22694
Fusarium graminearum 852
Botrytis cinerea 9835
Cochliobolus lunatus 146924
Mycosphaerella graminicola 72861
Zymoseptoria pseudotritici 799457
Talaromyces stipitatus 2483
Penicillium chrysogenum Wisconsin 143472
Penicillium subrubescens 6215
Cochliobolus lunatus 116911
Fusarium graminearum 3310
Talaromyces stipitatus 10359
Penicillium chrysogenum Wisconsin 142740
Penicillium subrubescens 11943
Aspergillus nidulans 2057
Aspergillus fumigatus 336
Aspergillus oryzae 3554
Aspergillus japonicus 42459
Aspergillus niger 10496
Pseudomonas fluorescens Vdh
Sphingomonas paucimobilis LigV
0,20

S2

Fig. S3. Maximum likelihood (ML; 500 bootstraps) phylogenetic tree of *A. niger* VdhA compared to selected fungal genomes. The scale bar shows a distance equivalent to 0.2 amino acid substitutions per site. Values over 50% bootstrap support are shown with ML values in black, Neighbor Joining values in purple and Minimum Evolution values in blue. Characterized enzymes are in bold. Blue font represents ascomycete fungi, red font basidiomycete fungi, green font bacteria, and orange font Saccharomycetes. Fungal species names are followed by protein IDs from JGI (http://genome.jgi-psf.org/programs/fungi/index.jsf).

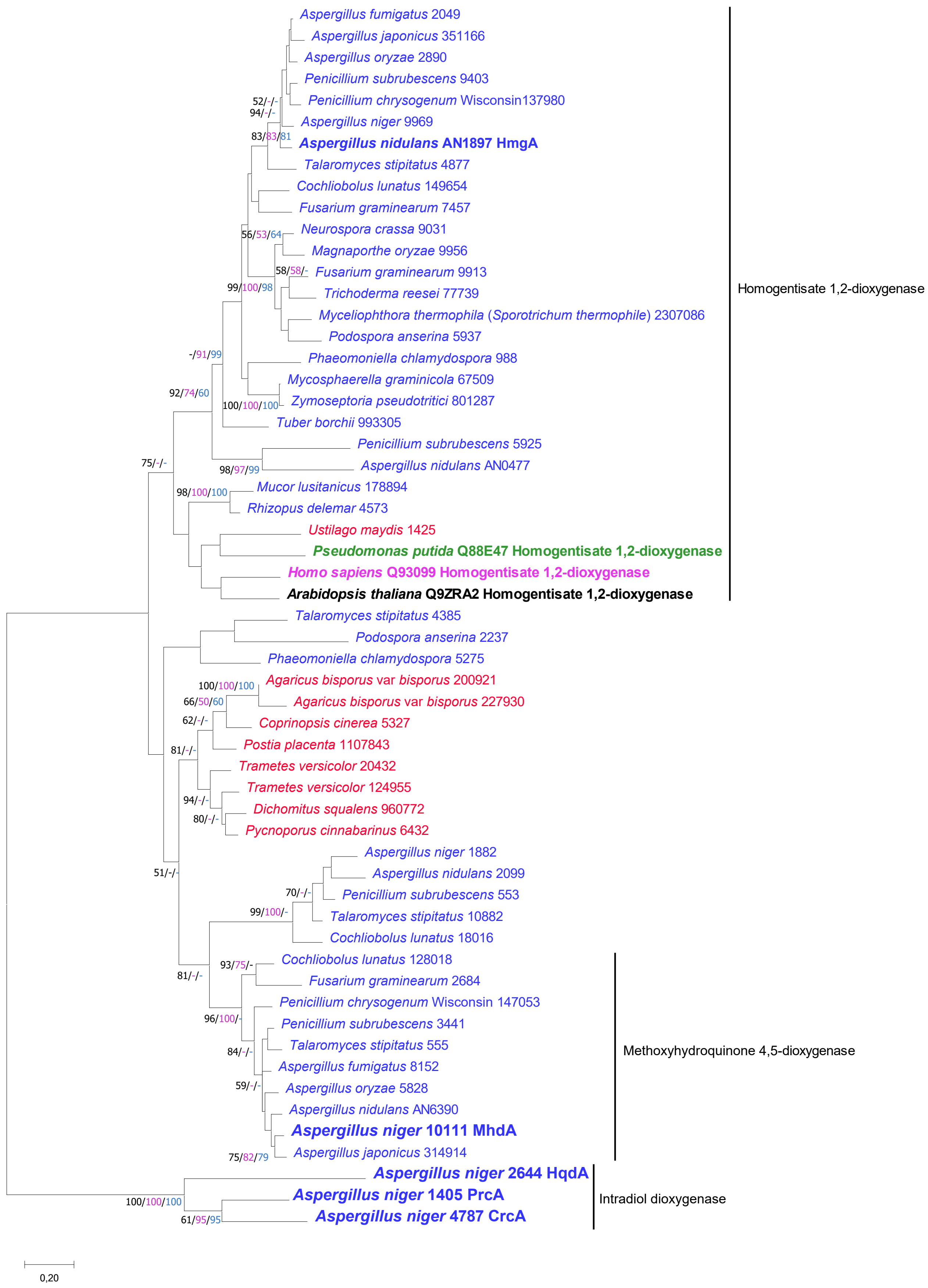

Fig. S4. Maximum likelihood (ML; 500 bootstraps) phylogenetic tree of *A. niger* MhdA compared to selected fungal genomes. The scale bar shows a distance equivalent to 0.2 amino acid substitutions per site. Values over 50% bootstrap support are shown with ML values in black, Neighbor Joining values in purple and Minimum Evolution values in blue. In bold are characterized enzymes. Blue font represents ascomycete fungi, red font basidiomycete fungi, green font bacteria, black font plants and pink font *Homo sapiens.* Fungal species names are followed by protein IDs from JGI (http://genome.jgi-psf.org/programs/fungi/index.jsf).

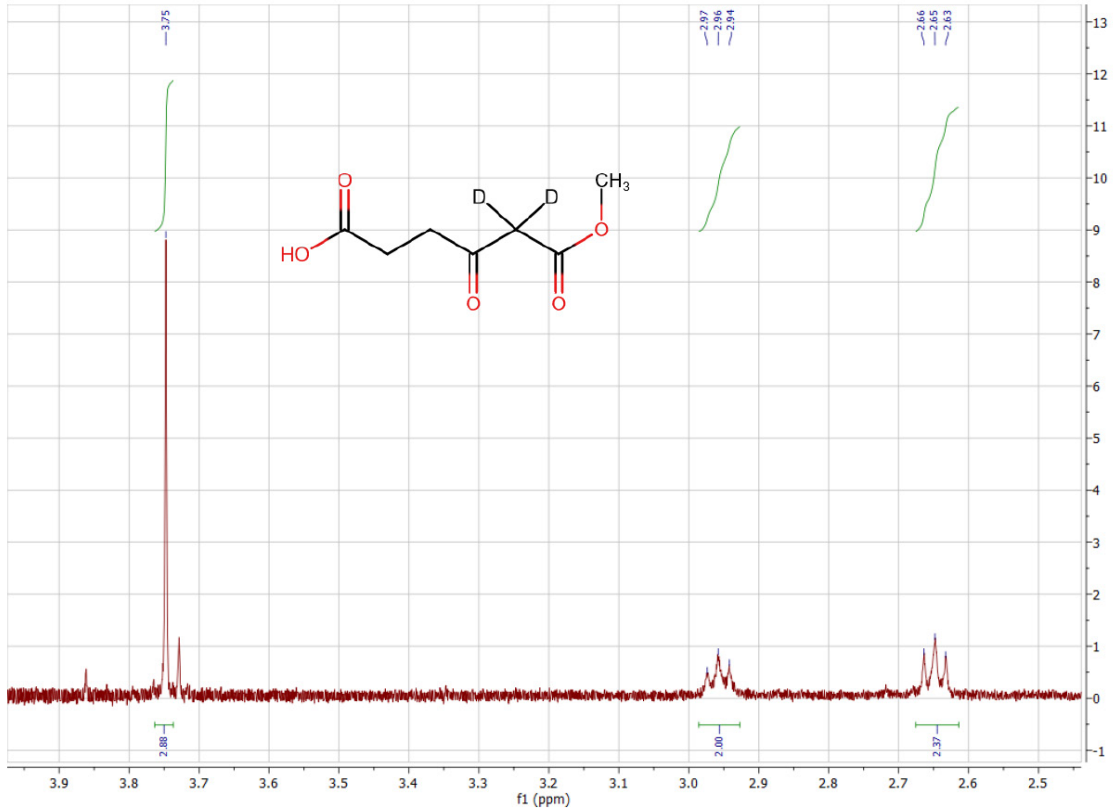

Fig. S5. 1H-NMR spectrum of 4-oxo-monomethyl adipate (D2O). The structure corresponds to 4-oxo-monomethyl adipate with D2 at C5 position.
